# Identification of Prognostic and Tumor Microenvironment by Shelterin Complex-Related Signatures in Oral Squamous Cell Carcinoma

**DOI:** 10.1155/2022/6849304

**Published:** 2022-06-15

**Authors:** Suwei Zhang, Hanhui Yu, Jiazhen Li, Liang Zhao, Lei Tan, Qiang Song, Cong Sun

**Affiliations:** ^1^Department of Clinical Laboratory, Shantou Central Hospital, Shantou, Guangdong Province 515041, China; ^2^Department of Neurosurgery, Shantou Central Hospital, Shantou, Guangdong Province 515041, China; ^3^Department of Stomatology, The First Affiliated Hospital of Xi'an Jiaotong, Xi'an, Shanxi Province 710061, China; ^4^Department of Structural Heart Disease, The First Affiliated Hospital of Xi'an Jiaotong University, Xi'an, Shanxi Province 710061, China

## Abstract

Oral squamous cell carcinoma (OSCC) is a common malignant tumor of the oral cavity. Shelterin complex gene (SG) has an important role in regulating telomere structure and length. SG is considered promising as a novel prognostic marker for cancer and a potential target for tumor therapy. However, SGs have not been systematically studied in OSCC. We analyzed SGs based on public data from OSCC patients and showed that SGs are closely associated with the prognosis of OSCC patients. Two different subtypes of SGs were identified in the TCGA and GEO cohorts, and LASSO regression analysis was used to further construct an SGs-related prognostic model. Randomized cohorts and different clinical subgroups validated the model's accuracy. The assessment of clinical characteristics, tumor mutational burden (TMB), and tumor microenvironment (TME) between high- and low-risk scores groups showed lower TMB, more abundant immune cell infiltration, and better prognosis in the low-risk group. According to the IPS analysis, patients in the low-risk group were more responsive to immunotherapy. This study establishes a foundation for research on SG and confirms that risk scores can predict prognosis and guide clinical treatment in OSCC patients.

## 1. Introduction

Telomeres are specialized nucleotide arrays located at the ends of linear chromosomes. As a result of shelterin being able to lengthen telomeres and protect the ends, malignant cells have bypassed senescence, which results in genomic instability [1–3]. The DNA damage signaling pathways inhibited by shelterin include classical NHEJ, ATM and ATR signaling, alternate NHEJ, resection, and homologous recombination. There are six proteins in the shelterin complex, including TERF2-interacting protein 1 (TERF2IP), TERF1 and TERF2 (telomeric repeat-binding proteins), adrenocortical dysplasia protein homolog (ACD), POT1 (patent for telomere protection), and TIF2 (TERF1-interacting protein 2) [4–6].

One of the most common oral cancers is OSCC (oral squamous cell carcinoma), with more than 350,000 diagnoses each year, corresponding to roughly 2% of all tumor diagnoses [7, 8]. OSCC patients have a low 5-year survival rate (less than 60%), and there are no optimal clinical treatment options [9, 10]. Therefore, novel treatment targets are urgently needed to improve. A more reliable prognostic model is also needed to make treatment more feasible.

Shelterin complex genes (SGs) have been implicated in cancer development in previous studies [11, 12]. Li et al. found that telomere dysfunction and cellular senescence could be induced by targeting POT1 with MiR-185 [13]. During gallbladder cancer development, the telomere length of the SG is also significantly altered [14]. SG is considered promising as a novel prognostic marker for cancer and a potential target for tumor therapy. In OSCC, its role is not yet fully understood. We must comprehensively evaluate immunocellular infiltration characteristics in the SG-regulated tumor microenvironment (TME) in order to better understand the microenvironment of OSCC and to develop personalized treatments.

In order to predict prognosis and guide treatment, it was of primary importance to comprehensively evaluate the expression patterns of SGs in OSCC and develop a prognostic risk scoring model for SGs. Risk scores were used to assess tumor mutational burden, tumor microenvironment, immunotherapy response, drug sensitivity, and clinical prognosis in OSCC patients. Findings from these studies may provide new perspectives on how to better understand and treat SGs in OSCC.

## 2. Methods

### 2.1. OSCC Data Preparation

OSCC patient-related data were retrieved from TCGA (https://portal.gdc.cancer.gov/) and GEO databases (https://www.ncbi.nlm.nih.gov/geo/). TCGA-OSCC data (workflow type, HTSeq-FPKM) were obtained from the TCGA-HNSCC project. In the subsequent analysis, FPKM values were transformed using log2 (FPKM +1) [15, 16]. Gene Expression Omnibus data for GSE41613 was obtained from (GEO, https://www.ncbi.nlm.nih.gov/geo/query/acc.cgi?acc=GSE41613) database, Platform: GPL570. An RMA normalization was carried out on the GSE41613 datasets. From GSE103322, we derived the data on single cells RNA-seq from oral cancer (http://www.ncbi.nlm.nih.gov/geo/query/acc.cgi?acc=GSE103322). Cells from 18 patients with oral tumors made up 5902 single cells [17]. Public databases listed above are freely accessible, and the study followed their publication guidelines and data access policies.

### 2.2. Identification of Differentially Expressed SGs

By analyzing TCGA-OSCC and GSE41613 samples, we aimed to determine which SGs differed in expression between normal and tumor tissues. The SVA package was used to normalize RNA expression profiles and eliminate batch effects on TCGA and GEO data [18]. Researchers have used the R package “limma” to identify SGs that show a significant difference (*P* > 0.05) between normal tissue and tumor tissue [19]. The Metascape database (https://metascape.org/gp/index.html#/main/step1) contains gene annotations and analyses [20]; this study used Metascape to perform enrichment analyses on SGs.

### 2.3. SGs-Based Classifications of OSCC Patients in the TCGA-OSCC and GSE41613 Cohorts

We identified the distinct patterns of SG expression in OSCC patients by using consensus clustering based on their expression levels to classify them further. The above steps were performed through the R package “ConsensusClusterPlus” and repeated 1000 times to ensure clustering stability [21, 22]. The CDF curve for the consensus heat map is determined by the area's relative change and the consensus heat map's consensus score. To determine the prognosis of patients with different OSCC, a Kaplan-Meier survival analysis was performed.

### 2.4. Gene Set Variation Analysis (GSVA)

Functional enrichment analysis of SGs clusters was conducted using the “GSVA” R package [23]. ClusterProfiler was used for functional annotation, and the MSigDB gene set file obtained from the GSEA-MSigDB database (https://www.gsea-msigdb.org/) was used to obtain the gene set (c2.cp.kegg.v7.2.symbols.gmt).

### 2.5. Construction of an SG-Related Risk Scores Model

SG-related patterns are differentiated by differently expressed genes (DEGs). Subsequently, DEGs with P < 0.05 were also included in the univariate Cox regression test utilizing the selection operator (LASSO) algorithm for dimensionality reduction and least absolute shrinkage [24–26]. Based on standardized expression levels and coefficients, each patient was assigned a risk score (RS). A median risk score was used to group patients into high- and low-risk groups. Using the R package “survminer,” survival analysis was conducted between groups of high- and low-risk individuals. Analyses of multivariate and univariate Cox regression were also performed to determine the prognostic value of risk scores.

### 2.6. Tumor Mutation Burdens (TMB)

In order to summarize OSCC patients' mutations, we used the COSMIC (Catalogue of Somatic Mutations in Cancer, https://cancer.sanger.ac.uk/cosmic) and then gathered genomic mutation data of TCGA-OSCC for further analysis [27, 28].

### 2.7. Evaluation of the Chemotherapy and Immunotherapy Response Based on RS

For predicting the IC50 of chemotherapy drugs for OSCC patients from TCGA, the “pRRophetic” package was used to explore their sensitivity to different treatments. We compared the high-risk and low-risk groups. The “ESTIMATE” package was used to calculate the stromal scores, ESTIMATE scores, immune scores, and tumor purity. Additionally, we assessed immune cell infiltration levels in the TME by using the ssGSEA algorithm in the R “GSVA” package. From The Cancer Immunome Atlas (TCIA), we downloaded immunophenotype scores (IPS) from the TCGA-OSCC project. The higher the IPS, the better the accuracy of the more accurate result [29, 30].

### 2.8. Statistical Analysis

Statistical analyses were carried out using R version 4.1.0, GraphPad Prism 8, and SPSS 23.0. We used the cluster profile package to examine GO, KEGG, and functional annotation enrichment. The ROC curve analysis was conducted using the “timeROC,” “survminer,” and “survival” R packages. The volcano and heat map were developed by R software's “ggplots” package. *P* values for all statistical analyses were two-sided, and a significance level of *P* < 0.05 was considered.

## 3. Results

### 3.1. Identification of SGs between Normal and Tumor Tissues in OSCC

**Figure 1:**
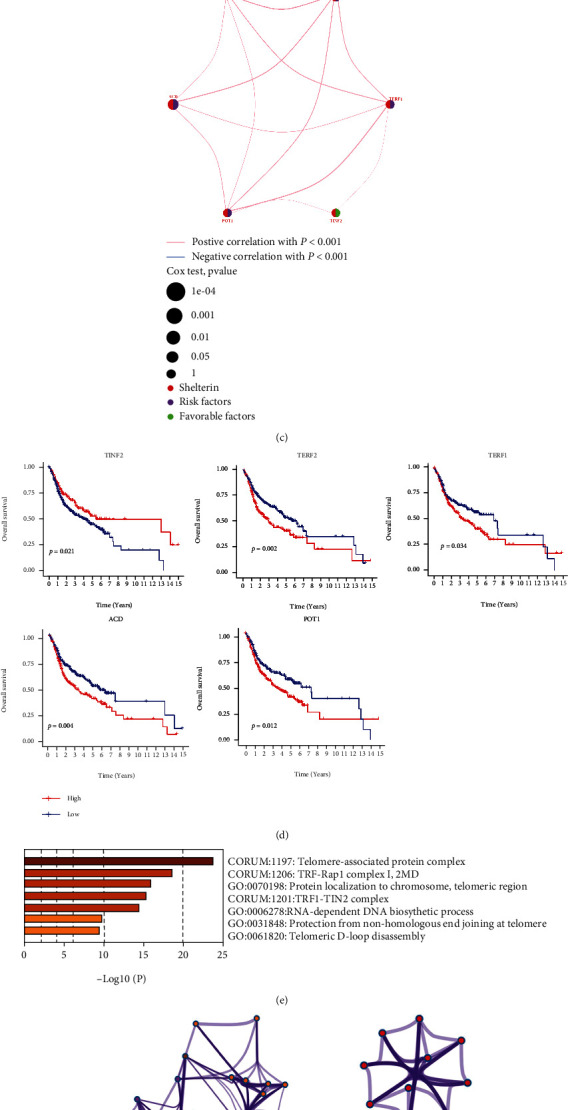
SGs in OSCC have distinct characteristics and differences. (a) Schematic illustration of shelterin complex. The shelterin complex is composed of six core proteins, including TERF2-interacting protein 1 (TERF2IP), telomeric repeat-binding factors 1 and 2 (TERF1 and TERF2), adrenocortical dysplasia protein homolog (ACD), protection of telomeres 1 (POT1), and TERF1-interacting protein 2 (TINF2). (b) The mRNA expression levels of SGs were compared between normal and tumor samples. ∗*P* < 0.05, ∗∗*P* < 0.01, and ∗∗∗*P* < 0.001. (c) A network of correlations including SGs. The lines connecting SGs represented their interaction with each other. The size of each circle represented the prognosis effect of each regulator and scaled by *P* value. Favorable factors for patients' survival were indicated by a green dot in the right half of the circle and risk factors indicated by the purple dot in the right half of the circle. (d) The results of survival analysis showed the relationship between the expression level of SGs and the prognosis of OSCC patients. (e) The image showed the histogram of the enriched pathways associated with the SGs. The abscissa was the value of -Log10P and longitudinal which were different enrichment pathways, sorted by the value of -Log10P. (f) The image showed the network of enriched terms. Each node represented an enriched term and was colored by its cluster ID.

The shelterin complex consists of six proteins (Figure 1(a)), termed TRF1, TRF2, RAP1, TPP1, POT1, and TIN2, and abnormal expression of shelterin has been observed in various types of cancers. However, the study of shelterin genes in OSCC is unclear. Compared with normal tissues, we found that all 6 SGs were significantly highly expressed in the tumor group (Figure 1(b)). The comprehensive landscape of the interactions between 6 SGs in OSCC patients is illustrated in the network. A positive correlation was found between the 6 SGs, many of which were risk factors for OSCC (Figure 1(c)). The occurrence and development of OSCC may be influenced by crosstalk. According to the survival analysis, high expression levels of TERF1, TERF2, ACD, and POT1 contributed to poor prognosis, while high expression level of TINF2 had a better survival advantage (Figure 1(d)). The functional enrichment analysis of SGs was performed through the Metascape website, and the results showed that SGs were mainly enriched in telomere-related pathways, such as telomere-associated protein complex, protection from nonhomologous end joining at telomere, RNA-dependent DNA biosynthetic process, and telomeric D-loop disassembly (Figure 1(e); Table 1). Enriched terms were selected and rendered as a network plot (Figure 1(f)).

### 3.2. Analysis of the Expression Distribution of SGs Based on Single-Cell Data

**Figure 2:**
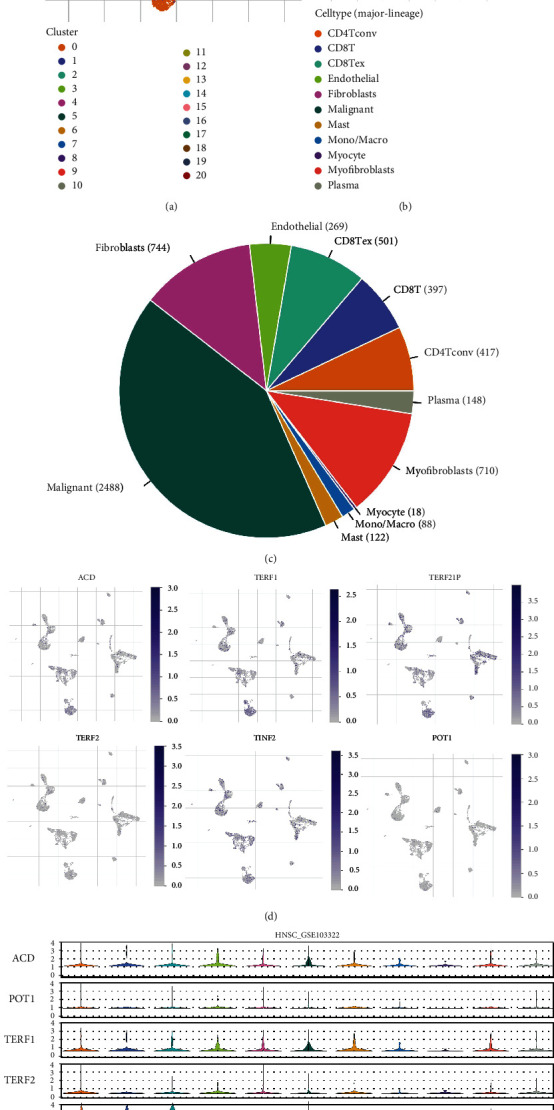
Analysis of the expression distribution of SGs based on single-cell data. The overview tab of the GSE103322 dataset. Two UMAP plots with cells colored by cluster ID (a) and cell type (b) are displayed. (c) The pie plot shows the cell number distribution of each cell type. (d) The expression of SGs is visualized at single-cell and cell-type resolution. (e) Violin plots visualize the distribution of SGs across different cell types.

We obtained 5902 single-cell data from 18 oral tumor patients from GSE103322 to determine the expression distribution of SGs in different cell types. Using TISCH (Tumor Immune Single-cell Hub) database, we visualized them. Findings revealed that these cells were divided into 20 clusters (Figure 2(a)). Primary cell types include CD4Tconv, CD8T, CD8Tex, endothelial, fibroblasts, malignant, Mast, mono/macro, myocyte, myofibroblasts, and plasma (Figure 2(b)). Among them, malignant enrichment was the most, and fibroblasts in the second (Figure 2(c)). Then we further visualized the expression levels of SGs at single-cell resolution (Figure 2(d)), and we found that TERF2I1 and TINF2 gene expression distribution were the most enriched, mainly distributed on CD8Tex, endothelial, fibroblasts, Mast, mono/macro, and myocyte (Figure 2(e)).

### 3.3. Classification of Tumors Based on SGs

**Figure 3:**
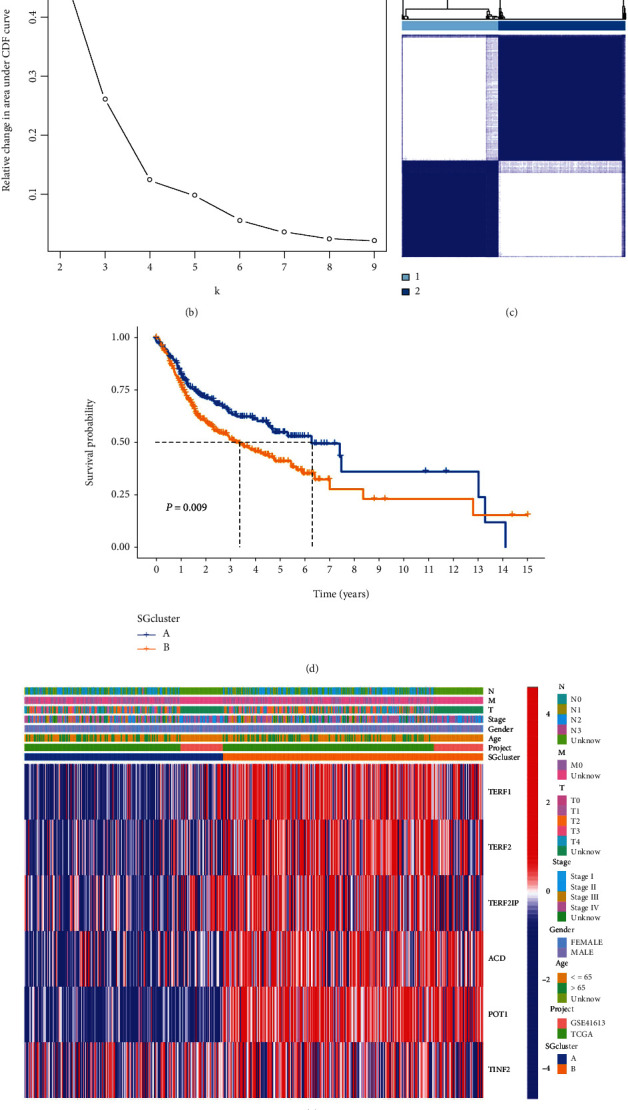
Subgroups of OSCC defined by genes involved in pyroptosis. (a) CDF curves of the consensus score (*k* = 2–9) in the two cohorts. (b) Relative change in the area under the CDF curve (*k* = 2–9) in the two cohorts. (c) Patients in two cohorts were grouped into two clusters according to the consensus clustering matrix (*k* = 2). (d) Kaplan–Meier survival analyses of the patients with SGCluster A and SGCluster B. (e) The two cluster heat maps based on SGs with clinical characteristics. Unknown: data not available. (f) PCA plot for OSCC patients based on the SGCluster.

**Figure 4:**
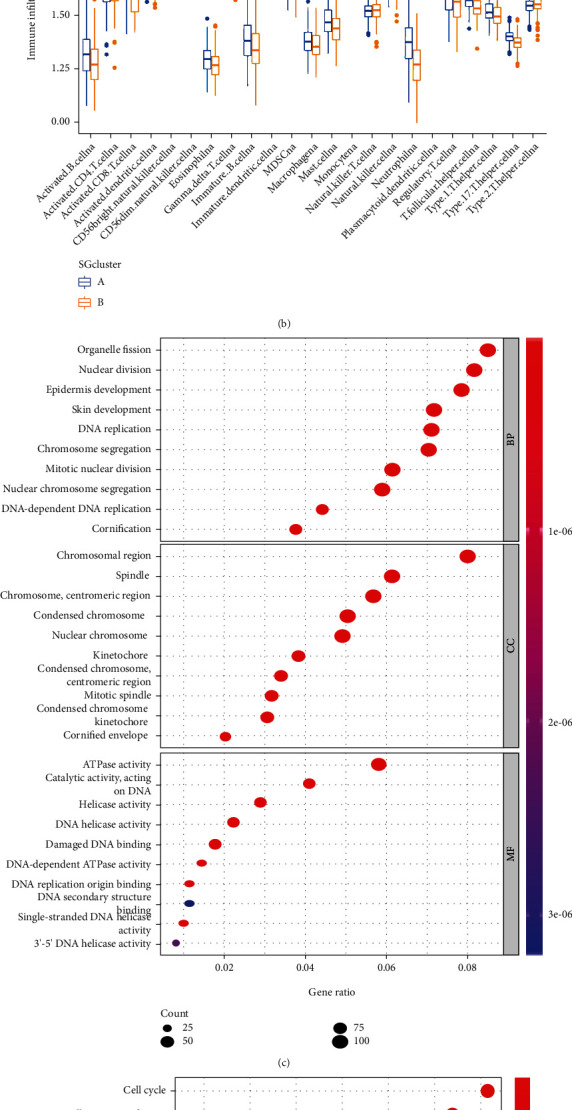
Different analyses between SGCluster A and SGCluster B. (a) Heat mapping was used to visualize the biological process by GSVA analysis in the 2 clusters. (b) Analysis of immune infiltrating cells between SGCluster A and SGCluster B. ∗*P* < 0.05, ∗∗*P* < 0.01, and ∗∗∗*P* < 0.001. GO (c) and KEGG (d) enrichment analysis of DEGs between SGCluster A and SGCluster B.

Based on the expression levels of SGs, we conducted a consensus clustering analysis on OSCC patients to better understand the role of SGs in OSCC. When we increased the clustering variable (*k*) from 2 to 9, we found the highest correlation between OSCC patients and other groups when *k* = 2 (Figures 3(a)–3(c)). Kaplan-Meier analysis revealed that SGCluster A had a significantly greater overall survival time (OS) than SGCluster B (*P* = 0.009, Figure 3(d)). Our heat map compares the SGs expression and clinical characteristics between the two clusters. Based on the heat map, we found that except for TINF2, the rest of the SGs were significantly enriched in SGCluster B (Figure 3(e)). The PCA analysis demonstrated that the SGs-based classification pattern could classify OSCC patients into two distinct subgroups (Figure 3(f)). The GSVA enrichment analysis explored the biological differences between these two clusters (Figure 4(a)). SGCluster A is significantly enriched for cancer and immune-related pathways, such as complement and coagulation cascades, autoimmune thyroid disease, ppar signaling pathway, olfactory transduction, cytokine receptor interaction, hematopoietic cell lineage, neuroactive ligand-receptor interaction, arachidonic acid metabolism, linoleic acid metabolism, and retinol metabolism. SGCluster B is enriched in nucleotide excision repair, cell cycle, glycosylphosphatidylinositol GPI anchor biosynthesis spliceosome, basal transcription factors, mismatch repair, aminoacyl-tRNA biosynthesis, homologous recombination, ubiquitin-mediated proteolysis, and RNA degradation. We also analyzed the level of immune infiltrating cells between the two subgroups (Figure 4(b)). We found that SGCluster A exhibited a greater enrichment of immune cells, such as activated B cells, CD8 T cells, eosinophilia, macrophage, Mast cell, and neutrophil, which may also be the one reason why SGCluster A has a better prognosis.

In addition, we identified 1329 DEGs between SGCluster A and SGCluster B (Supplementary file 1) and performed functional enrichment analysis on them. The DEGs enriched in GO pathways were mainly involved in DNA replication, DNA helicase activity, condensed chromosome, nuclear chromosome, kinetochore, and DNA replication (Figure 4(c)). In the KEGG enrichment analysis, DEGs were mostly enriched in pathways related to cell cycle, DNA replication, and cellular senescence, such as cell cycle, cellular senescence, ECM-receptor interaction, base excision repair, p53 signaling pathway, and PI3K-Akt signaling pathway (Figure 4(d)).

### 3.4. Development and Validation of an SGs-Related Risk Signature

**Figure 5:**
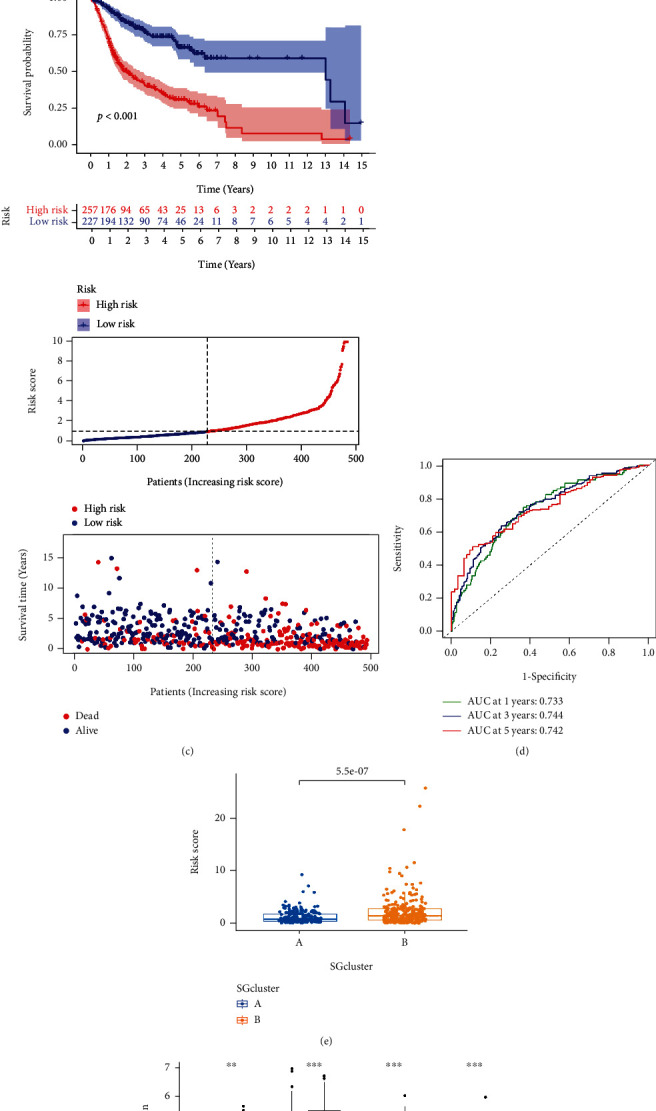
Construction of SGs-related risk signature. (a) Validation was performed for tuning parameter selection through the least absolute shrinkage and selection operator (LASSO) regression model for overall survival (OS). (b) Cross-validation for tuning parameter selection in the lasso regression. (c) SGs-related prognostic model constructed in total OSCC patients. (d) Plots of the AUC for time-dependent ROC performance. (e) SGCluster B has a higher risk score. (f) Expression levels of SGs genes in high- and low-risk groups.

Based on DEGs between SGCluster A and SGCluster B, we developed a prognostic model to explore further the application of SGs in OSCC patients' prognosis and treatment. To screen DEGs for genes associated with prognosis, we performed a univariate Cox analysis (Supplementary file 2). Based on the results of the LASSO algorithm, the best prognostic genes were identified in OSCC patients (Figures 5(a) and 5(b)). We constructed multivariate Cox prediction signatures based on the prognostic genes identified with LASSO. The final analysis identified 14 genes associated with risk. Patients with OSCC were classified into low- and high-risk categories based on their median risk score. According to Kaplan-Meier plots, low-risk patients tend to have a better prognosis (Figure 5(c)). We constructed time-dependent ROC curves to evaluate the model's predictive ability, with AUCs reaching 0.733 after 1 year, 0.744 after 2 years, and 0.742 after 5 years (Figure 5(d)). The results indicate that the model is a good predictor. In addition, we evaluated the risk scores of SGCluster A and SGCluster B. We found that SGCluster A has a lower risk score (Figure 5(e)), supporting our previous findings that SGCluster A has a better outcome (Figure 3(d)).  Figure 5(f) shows the expression levels of specific SGs in high-risk and low-risk groups, in which TERF1, TERF2, ACD, and POT1 are all significantly highly expressed in the high-risk population (Figure 5(f)).

**Figure 6:**
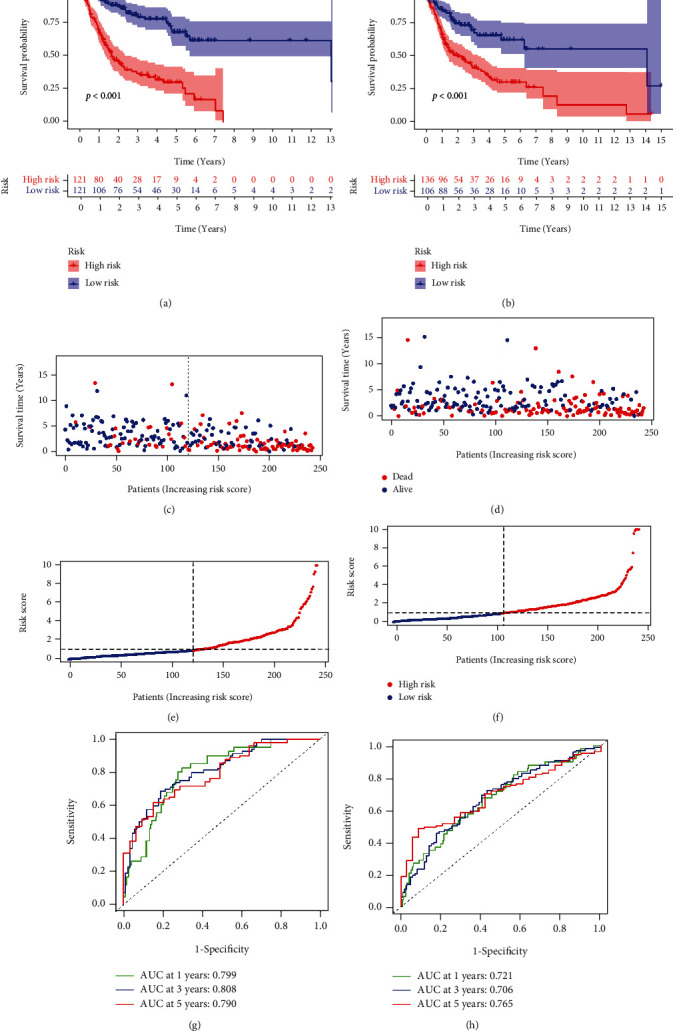
Verification of SGs-related risk signatures. Validation cohort 1 (a, c, e, and g), and the validation cohort 2 (b, d, f, and h). (a, b) Kaplan–Meier survival curve. (c, d) Survival statuses of high-risk and low-risk patients. (e, f) The distribution of the risk scores. (g, h) Plots of the AUC for time-dependent ROC performance.

To further test the model's robustness, OSCC patients were randomly assigned to validation cohort 1 (Figures 6(a)–6(c), and 6(e)) and validation cohort 2 (Figures 6(b)–6(d), and 6(f)). We calculated the risk score using the same algorithm, and we found that greater risk was associated with a worse outcome (Figures 6(a) and 6(b)). The AUC confirmed the reliability of the model results for both the validation cohort 1 (Figure 6(g); AUC at one, three, and five years were 0.799, 0.808, and 0.790, respectively) and the validation cohort 2 (Figure 6(h); AUC at 1, 3, and 5 years were 0.721, 0.706, and 0.765, respectively).

### 3.5. Relationship between Risk Scores and Clinical Characteristics

**Figure 7:**
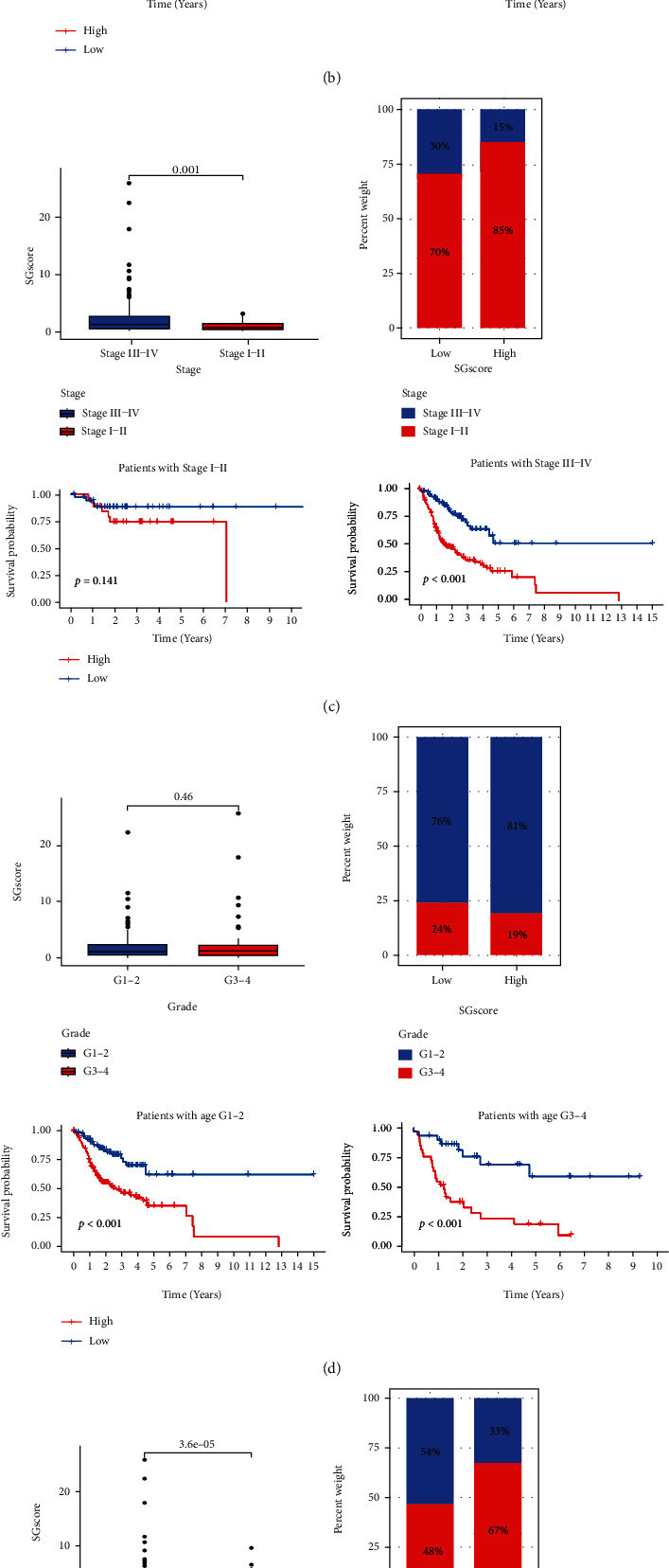
Relationship between risk scores and clinical characteristics. Relationship between risk score and age (a), gender (b), stage (c), grade (d), T (e), and N (f).

**Figure 8:**
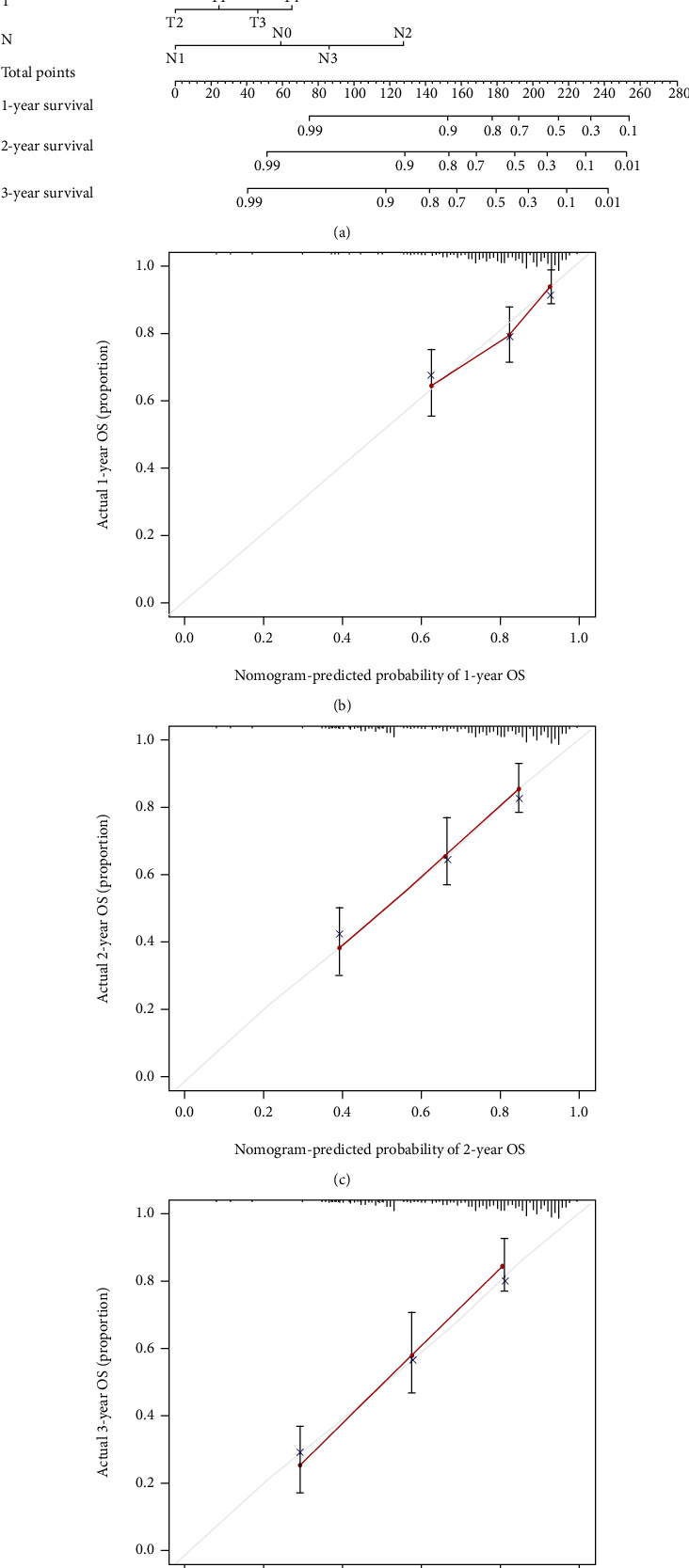
Nomogram to predict the 1-year, 2-year, and 3-year overall survival rates of OSCC patients. (a) A nomogram for predicting survival. Nomogram calibration plots for predicting OS at 1 (b), 2 (c), and 3 (d) years.

We analyzed the relationship between clinical characteristics (age, gender, stage, grade, T, and N) and risk scores to validate the accuracy of risk scores further and identify their role (Figures 7(a)–7(f)). A significant association was found between risk scores and stage (Figure 7(c)), T stage (Figure 7(e)), and N stage (Figure 7(f)), and patients with poorer clinical characteristics (stages III-IV, N1-3) tended to have higher risk scores. In addition, we found that risk score remained an excellent predictor (patients with age ≤65, age >65, MALE, FEMALE, stages III-IV, G1-2, G3-4, T1-2, T3-4, N0, and N1-3). We created risk scores as well as nomograms to extend their clinical application. Each patient was assigned a total score based on the scores of the item indicators, and patients with higher total scores had poorer clinical outcomes (Figure 8(a)). Calibrating the nomogram was good predictive power (Figures 8(b)–8(d)).

### 3.6. SGs-Related Risk Scores Could Predict and Represent Tumor Mutational Burden (TMB)

**Figure 9:**
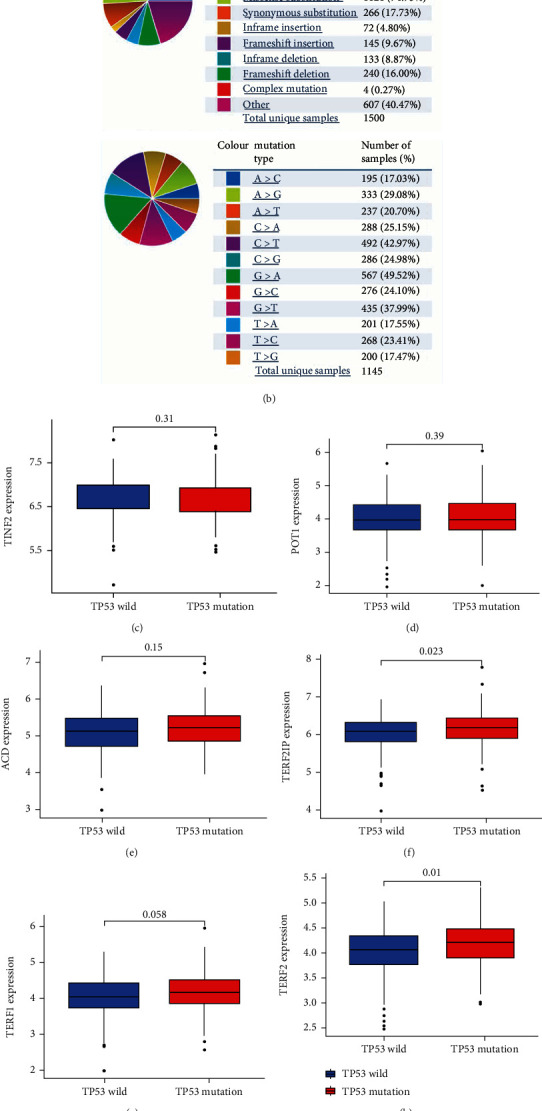
Relationship between risk score and TMB. COSMIC database analysis of OSCC mutation distributions (a) and its types (b). (c–h) Correlation analysis of TP53 mutation and gene expression level of SGs. (i) High TMB scores are associated with poorer outcomes. (j) Distribution of TMB in high- and low-risk score groups.

According to an increasing amount of evidence, TMB may be a catalyst for tumor progression. Using the COSMIC database, we analyzed the mutation status of OSCC. The most common mutation types in OSCC were missense, G > A, and C > T mutations (Figures 9(a) and 9(b)). According to the TCGA data, TP53 is the gene with the highest mutation rate in OSCC, so we looked into the relationship between TP53 mutations and gene expression levels in this tumor (Figures 9(c)–9(h)), determining that the TP53 mutation group has a higher level of TERF2IP (Figure 9(f)) and TERF2 (Figure 9(h)) expression. In addition, high TMB scores are associated with a worse prognosis (Figure 9(i)). This resulted in a higher TMB score in the high-risk group (Figure 9(j)), indicating the importance of the risk score in TMB.

### 3.7. The Role of Risk Scores in the Tumor Microenvironment (TME)

**Figure 10:**
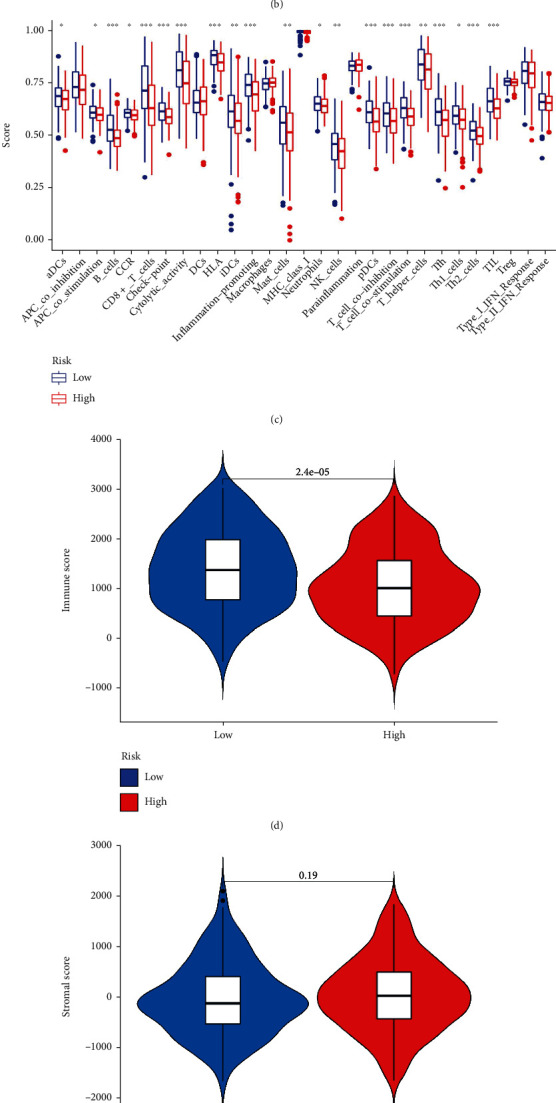
The relationship between risk score and TME. (a) Correlation analysis of immune infiltrating cells and risk score. (b) Heat map of the relationship between microenvironment and risk score. (c) Enrichment analysis of immune cells and immune-related pathways in high- and low-risk groups. ∗*P* < 0.05, ∗∗*P* < 0.01, and ∗∗∗*P* < 0.001. Comparison of immune score (d), stromal score (e), tumor purity (f), and ESTIMATE score (g) in high- and low-risk groups.

The application of the ssGSEA algorithm allowed us to estimate immune cell infiltration in the high-risk and low-risk groups. Results revealed that the low-risk group had more immune cells present (Supplementary Figure 1); the resting Mast cells, CD4 memory activated T cells, CD8 naive B cells, T cell regulatory (Tregs) cells, plasma cells, and T cell CD8 were all negatively correlated with risk. The risk scores were positively correlated with macrophages M0, CD4 memory resting T cells, NK cells resting, and Mast cells activated (Figure 10(a)). Furthermore, we used ESTIMATE (Figures 10(d)–10(g)). ssGSEA algorithms (Figure 10(c)) were used to analyze the TME of OSCC. The low-risk group had a higher immune score, immune-infiltrating cells, and immune pathways (Figure 10(d)).

### 3.8. Exploring the Application Value of Risk Score in Clinical Treatment

**Figure 11:**
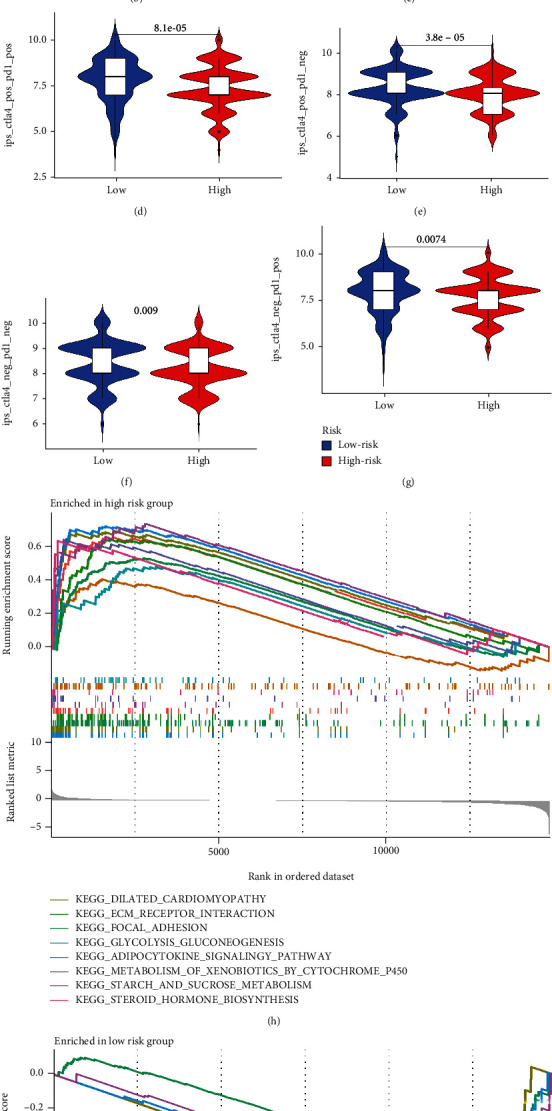
Application of risk score in clinical treatment. (a) The risk score is an independent prognostic indicator in OSCC patients. (b) Expression levels of PD1 in high- and low-risk groups. (c) Expression levels of CTLA4 in high- and low-risk groups. (d) CTLA4+PD1+. (e) CTLA4+ PD1−. (f) CTLA4− PD1−. (g) CTLA4− PD1+. (h) GSEA enrichment results for the high-risk group. (i) GSEA enrichment results for the high-risk group.

**Figure 12:**
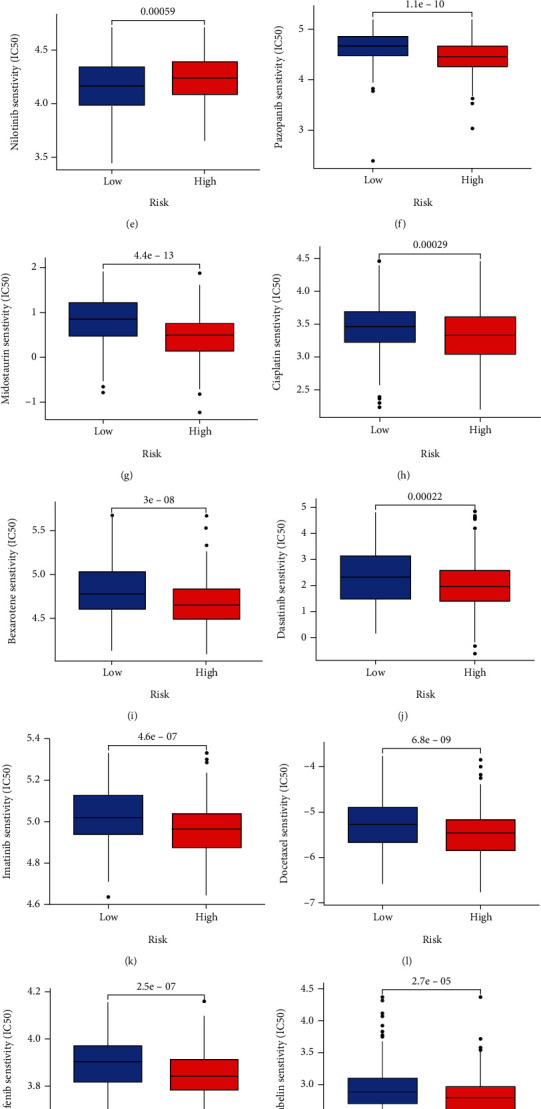
(a–p) Analysis of drug sensitivity in high and low risk groups.

Risk scores related to SGs have been found to play an important role in TMB and TME. In order to further explore the clinical utility of risk scores, we performed univariate and multivariate Cox analyses to identify their prognostic value. Based on the findings, OSCC patients' risk score was an independent prognostic factor (Figure 11(a)). A wide variety of tumors have been treated with immunotherapy. Through the TCIA database, OSCC patients' IPS data were examined to determine the risk score's role in immunotherapy (Figures 11(b)–11(g)). In low-risk patients, CTLA4 expression was significantly high (Figure 11(c)). Accordingly, the IPS score showed that low-risk patients received immunotherapy more readily than high-risk patients (Figures 11(d)–11(g)), which could help our clinical treatment. GSEA enrichment analysis revealed the high-risk groups tended to be enriched for the following: dilated cardiomyopathy, ECM receptor interaction, metabolism of xenobiotics by cytochrome p450, focal adhesion, adipocytokine signaling pathway, starch, sucrose metabolism, and steroid hormone biosynthesis (Figure 11(h)). Low-risk group members were primarily enriched for allograft rejection, an intestinal immune network for igan production, cell adhesion molecules, primary immunodeficiency, and autoimmune thyroid disease (Figure 11(i)). Based on the GDSC database, we also evaluated the response of chemotherapeutic agents in high and low patients (Genomics of Drug Sensitivity in Cancer, https://www.cancerrxgene.org/). Using ridge regression, we estimated the half-maximal inhibitory concentration (IC50) of samples. We calculated the prediction accuracy using the R package “pRRophetic” (Figures 12(a)–12(p)). In light of the above results, we believe that risk scores based on SGs may be useful for guiding clinical treatment.

## 4. Discussion

Tumor growth and metastasis are influenced by SGs that regulate viability, apoptosis, proliferation, adhesion, migration, and metastasis [31–33]. It has also been proposed that mutations in SGs may also be associated with the acquisition of somatic aberrations that accelerate cancer development [34–36]. More importantly, SGs promote or inhibit the growth of tumors by influencing the tumor compartment and its microenvironment. SGs have been extensively studied as therapeutic targets for cancer.

We examined the expression of SGs and their prognostic characteristics in OSCC patients, concluding that three of the six SGs were significantly elevated. Additionally, 4 were identified as OSCC risk factors (TERF1, TERF2, ACD, and POT1). As a result of the functional enrichment analysis, they were largely enriched in pathways related to telomeres (such as telomere-associated protein complex, protection from nonhomologous end joining at telomere, RNA-dependent DNA biosynthetic process, and telomeric D-loop disassembly). Several cancer types require telomere-associated proteins to maintain normal telomere function [33, 37, 38]. To further explore the role of SGs in OSCC, a consensus clustering analysis was conducted on OSCC patients based on their SG expression levels. We found that OSCC patients could be divided into two subgroups (SGCluster A and SGCluster B). Furthermore, SGCluster A has a higher enrichment of immune infiltrating cells, which may be one of the reasons for its better prognosis. Furthermore, we explored the differences between the two subgroups and identified 1329 DEGs. We identified DEGs based on GO and KEGG enrichment analysis, which, as expected, significantly correlated with pathways relating to cell cycle, senescence, apoptosis, chromosomes, and tumorigenesis development.

We developed prognostic models based on these DEGs. In order to expand the use of SGs in prognosis and clinical treatment for OSCC patients, we tested their stability and accuracy among randomized cohorts and different clinical subgroups. As a result, the model accurately predicted patients' prognoses. Notably, patients in the low-risk group tended to have lower TMB and more abundant immune-infiltrating cells, including Mast cells resting, T cells CD4 memory activated, T cells follicular helper, T cells regulatory (Tregs), plasma cells, T cells CD8, and B cells naive. Previous studies have suggested that highly abundant TME may be more sensitive to immunotherapy. In order to identify high- and low-risk immunotherapy groups, we calculated IPS scores. We found that low-risk groups tended to have higher IPS scores, representing the low-risk groups more sensitive to immunotherapy. The drug sensitivity analysis revealed that various chemotherapeutic drugs are more or less sensitive to high- and low-risk groups of patients. The findings of our study can help treat the OSCC patients and update the study of SGs. We believe that the risk scores can be used to predict OSCC prognosis and help predict patients' clinical response to immunotherapy.

However, there are several limitations to this study. The majority of the data we used came from public databases, so more experiments are required to verify the findings. In addition, studies reporting on SGs' role in OSCC are few, and our study only provides theoretical foundations for future experimental testing.

## 5. Conclusion

In summary, we systematically examined the expression levels and prognostic relevance of SGs in OSCC. In addition, a risk score model for SGs was constructed to evaluate the prognosis and TME of OSCC. Using risk scores, it is possible to predict OSCC prognosis and assess TME and immunotherapy responses. Risk scores allow for more individualized clinical treatment and will assist in guiding medical practice.

## Figures and Tables

**Table 1:** The functional enrichment analysis of SGs.

GO	Description	Count	Log10(P)	Log10(q)
CORUM:1197	Telomere-associated protein complex	6	-24.02	-19.99
CORUM:1206	TRF-Rap1 complex I, 2MD	5	-18.77	-15.38
GO:0070198	Protein localization to chromosome, telomeric region	5	-15.90	-12.94
CORUM:1201	TRF1-TIN2 complex	4	-15.36	-12.53
GO:0006278	RNA-dependent DNA biosynthetic process	5	-14.47	-11.72
GO:0031848	Protection from non-homologous end joining at telomere	3	-9.82	-7.26
GO:0061820	Telomeric D-loop disassembly	3	-9.44	-6.90

“Count” is the number of genes in the given ontology term. “Log10(P)” is the *P* value in log base 10. “Log10(q)” is the multitest adjusted *P* value in log base 10.

## Data Availability

The data sets analyzed during the current study are available in the TCGA (https://portal.gdc.cancer.gov/) and GEO repository (https://www.ncbi.nlm.nih.gov/geo/).

## Abbreviations

OSCC: Oral squamous cell carcinoma
GEO: Gene Expression Omnibus
TCGA: The Cancer Genome Atlas
OS: Overall survival
ROC: Receiver operating characteristic
TMB: Tumor mutational burden
TME: Tumor microenvironment
GO: Gene ontology
KEGG: Kyoto Encyclopedia of Genes and Genomes
DEGs: Differentially expressed genes
SGs: Shelterin complex genes.

## Supplementary Materials

Supplementary Materials Supplementary Figure 1: immune infiltrating cells in high and low risk groups. ∗*P* < 0.05, ∗∗*P* < 0.01, and ∗∗∗*P* < 0.001. Supplementary file 1: 1329 DEGs between SGCluster A and SGCluster B. Supplementary file 2: result of univariate Cox analysis.
