# Supplementary figures and images for: Identification of Prognostic and Tumor Microenvironment by Shelterin Complex-Related Signatures in Oral Squamous Cell Carcinoma
Source: Oxid Med Cell Longev. 2022 Jun 15;2022:6849304. doi: 10.1155/2022/6849304 (PMC9217620; doi:10.1155/2022/6849304)

risk low high

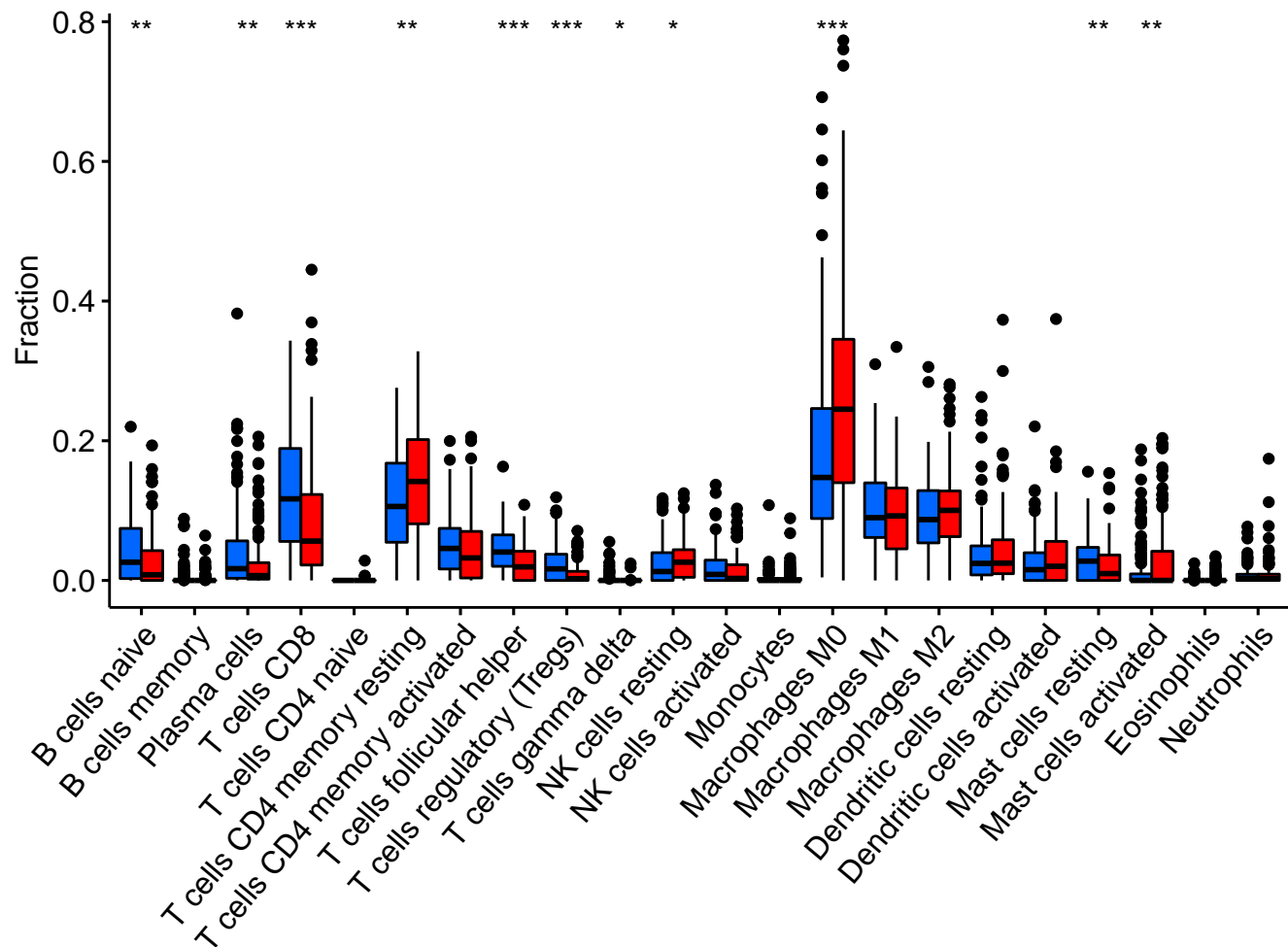

Supplement: Supplementary Materials — Supplementary Figure 1: immune infiltrating cells in high and low risk groups. ∗P < 0.05, ∗∗P < 0.01, and ∗∗∗P < 0.001. Supplementary file 1: 1329 DEGs between SGCluster A and SGCluster B. Supplementary file 2: result of univariate Cox analysis. [file 6849304.f1.zip › S1.pdf]
